# Humanized Mice for Infectious and Neurodegenerative disorders

**DOI:** 10.1186/s12977-021-00557-1

**Published:** 2021-06-05

**Authors:** Prasanta K. Dash, Santhi Gorantla, Larisa Poluektova, Mahmudul Hasan, Emiko Waight, Chen Zhang, Milica Markovic, Benson Edagwa, Jatin Machhi, Katherine E. Olson, Xinglong Wang, R. Lee Mosley, Bhavesh Kevadiya, Howard E. Gendelman

**Affiliations:** 1 Department of Pharmacology and Experimental Neuroscience, University of Nebraska Medical Center, Omaha, NE 68198 USA; 2 Department of Pharmaceutical Sciences, University of Nebraska Medical Center, Omaha, NE 68198 USA

## Abstract

Humanized mice model human disease and as such are used commonly for research studies of infectious, degenerative and cancer disorders. Recent models also reflect hematopoiesis, natural immunity, neurobiology, and molecular pathways that influence disease pathobiology. A spectrum of immunodeficient mouse strains permit long-lived human progenitor cell engraftments. The presence of both innate and adaptive immunity enables high levels of human hematolymphoid reconstitution with cell susceptibility to a broad range of microbial infections. These mice also facilitate investigations of human pathobiology, natural disease processes and therapeutic efficacy in a broad spectrum of human disorders. However, a bridge between humans and mice requires a complete understanding of pathogen dose, co-morbidities, disease progression, environment, and genetics which can be mirrored in these mice. These must be considered for understanding of microbial susceptibility, prevention, and disease progression. With known common limitations for access to human tissues, evaluation of metabolic and physiological changes and limitations in large animal numbers, studies in mice prove important in planning human clinical trials. To these ends, this review serves to outline how humanized mice can be used in viral and pharmacologic research emphasizing both current and future studies of viral and neurodegenerative diseases. In all, humanized mouse provides cost-effective, high throughput studies of infection or degeneration in natural pathogen host cells, and the ability to test transmission and eradication of disease.
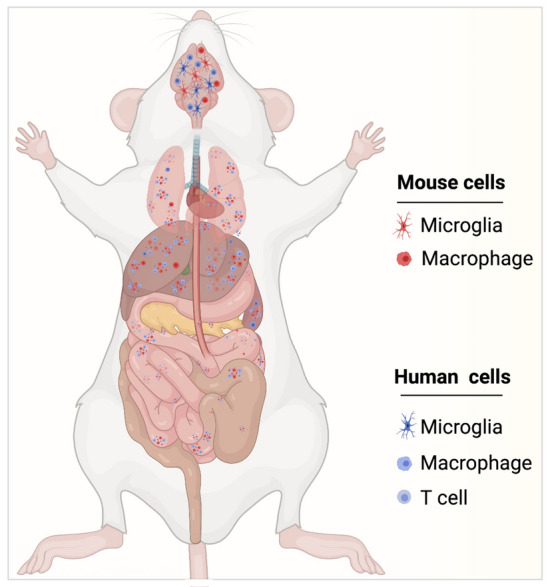

## Introduction

Rodents are the most common animal used in biomedical research laboratories. This is driven largely by low cost, small size, ease of housing, maintenance, large litter sizes and availability of inbred strains. For infectious diseases these animals can be used to study pathogen cell and tissue tropisms, replication, and virulence. Moreover, advances in disease pathogenesis, pharmacologic and vaccine research serves to mitigate the health burden of not simply infectious disease but also metabolic, cancerous, and degenerative disorders [1, 2]. Animal models used to study each disease independent of etiology must accurately reflect the clinical and pathological features of the human condition. When those features align, models become indispensable partners in research efforts aimed to better understand pathobiological mechanisms, and hence therapies deployed for translational preclinical investigations. Thus, the needs to better model human disease is essential to accelerate relevant pathogenic and treatment findings or strategies that can be translated to the clinic. The most applicable animal model of human disease closely recapitulates clinical symptoms and disease pathogenesis seen during the disease course. For infectious diseases in particular, the animal model should meet permissibility to the inciting pathogens with a clearly defined route of infection that parallels a susceptible human host. Such criteria are imperative for any United States Food and Drug Administration approvals, when and where vaccines and therapeutics cannot ethically be tested on humans. These enable final approvals which can only be made after preclinical tests are completed. The most relevant models’ rests in the field of infectious diseases, as many of the studied pathogens are human specific. To such ends, several studies of infectious pathogens can only be completed using humanized mice. Examples include studies of *Neisseria meningitides,* and when conducted in humanized mice display specificity to human microvessels and induce vascular leakage and tissue necrosis [3]. *Leishmania major* provides yet another example as infection can proceed in human macrophages with secondary adaptive T cell responses [4]. Human T cell leukemia virus (HTLV) demonstrate productive infection and expansion of virus specific CD4 + T cells [5]. Virus-specific immune responses have also been observed in these humanized mice. Dengue virus infection occurs in the spleen, bone marrow, and liver of humanized mice and these animals develop human disease-like signs and symptoms that include fever, apathy, rash, and weight loss [6, 7]. Likewise, Epstein Barr virus (EBV) or human herpes simplex virus type 4 (HHV-4) and its associated lymphoproliferative disorders and tumor development are reflected in humanized mice [8]. Kaposi’s sarcoma-associated herpesvirus, or HHV-8, leads to persistent latent infection of B cells and macrophages within spleen of humanized mice with viral dissemination to the skin [9]. HHV-2 infections show T and natural killer (NK) cell responses, antibody responses and ongoing viral replication in humanized mice [10]. Human cytomegalovirus (CMV) or HHV-5 can readily be detected in the liver, spleen, and bone marrow of humanized mice [11]. John Cunningham (JC) virus is well studied in humanized mice demonstrating peripheral and central nervous system infection [12, 13]. *Salmonella enterica*, the causative agent of typhoid fever in humans, can also be investigated in humanized mice [14]. Tuberculosis infections were mirrored in these mice and demonstrate CD4 + T cell and macrophage-dependent granuloma-like structure formation after infection [15]*.* Further treatment with cytokines like, granulocyte macrophage colony stimulating factor (GM-CSF) in these animals demonstrate infection control [15]. Other human diseases such as influenza, Ebola, Hanta virus pulmonary syndrome (HPS), malaria, and sepsis, have been studied using different models of humanized mice, and insights have been gained regarding their severity, transmission and therapeutic efficacy [16].

Other examples of viral and non-viral diseases studied in humanized mice include the human immunodeficiency virus type one (HIV-1) [17], severe acute respiratory syndrome coronavirus 2 (SARS-CoV-2) [18], influenza [19], Zika (ZIKV) [20], hepatitis C (HCV) [21], dengue viruses [19] and malaria [22, 23]. These studies were possible after the research community overcame the limitations imposed by grafting human tissues. Mice lacking a functional adaptive immune system such as the severe combined immunodeficient (SCID) or recombination activating gene 1 (RAG-1) knock-out become permissive to engraftment of human immune cells from human solid organ tissues or cord blood [24]. At the same time, removal of mouse genes like common gamma chain of the interleukin-2 receptor enabled the models to reflect multiple aspects of the human innate and adaptive immune response [16, 17, 25]. In the early 2000s, the development of immunodeficient mice bearing mutations in the IL-2 receptor gamma chain (*IL2rg*^*null*^) proved to be a breakthrough in humanized mouse development [26]. The common gamma chain (γ_C_) represents an important component of receptors for IL-2, IL-4, IL-7, IL-9, IL-15, and IL-21, and is crucial for the signaling of human cytokines. The attenuation of cell cytokine signaling pathways by γ_C_ which are involved in the survival, differentiation, and function of lymphocytes impairs the development of the mouse lymphoid system. In combination with either protein kinase DNA activated catalytic polypeptide mutation (*Prkdc*^*scid*^ or *scid*), or with *Rag* 1 or 2 (*Rag1*^*null*^* or Rag2 *^*null*^) mutations, adaptive immunity is depleted. These mice also exhibit deficiencies of innate immunity and lack murine NK cells [26].

New therapeutic agents and preventive strategies require in-depth understanding of disease pathobiology. Appropriate model systems are also required for testing the safety and efficacy of disease preventative measures [27, 28]. Selection of a model to mimic disease is driven by physiologic linkages to humans, ease of use, reproducibility, safety, and cost [27]. Due to limitations associated with non-human primates (NHPs) that include expense, availability, time, and genetic limitations, there is a need for small animal models as human surrogates [29, 30]. Rodent experiments can assess study reproducibility while controlling host genetics in response to the pathogen or to the disease [28]. Although medically relevant pathogens can cause disease in inbred mouse strains, pathogens such as ZIKA virus, measles virus, Middle East respiratory syndrome coronavirus (MERS-CoV), human norovirus, and Crimean-Congo hemorrhagic fever viruses do not produce disease in mouse strains [20, 31, 32]. Notably, the genetic differences between mice and humans interfere with a pathogen’s ability to elicit human-like disease outcomes in rodents [33–35]. To overcome these limitations, humanized mice were developed to study host–pathogen interactions. Herein, we focus first on new models of humanized mice then evaluate their use to study infectious, neurodegenerative, and inflammatory diseases and therapeutics. We also propose new models and extend the utility ranges of existing ones.

## Human cell-grafted mice

In cases where mice are not permissive to microbial infection an alternative is “genetically-modified” mice that can be made by the introduction of human-specific genes or engrafting human organs or cells [16, 25, 36, 37]. Mice cannot be used to study hepatitis B and C virus (HBV and HCV), herpes viruses and/or HIV-1 where several genes regulate host range, and thus preclude expression of factors that fully recapitulate and promote disease [38]. Another factor that limits the use of rodent models to recapitulate human disease is in differences between host immune responses [16]. This leads to limitations in engraftment efficiency with high rates of tissue rejection. Both reflect common deficiencies to fully recapitulate antigen-specific immune responses [16]. Despite such limitations, human intestinal xenografted mice have been used successfully to support *Entamoeba histolytica* infections [25, 39]. These model systems can facilitate studies of pathogen interactions with human cells and tissues [40] serving as important pre-clinical tools for biomedical research [24, 29, 30, 41]. As of today, the three most widely used immunodeficient strains are NOD.Cg-*Prkdc*^*scid*^*Il2rg*^*tm1Wjl*^ (NSG), NODShi.Cg*-Prkdc*^*scid*^*Il2rg*^*tm1Sug*^ (NOG), and C;129S4- *Rag2*^*tm1Flv*^*Il2rg*^*tm1Flv*^ (commonly referred to as BALB/c-*Rag2*^*null*^* IL2rg*^*null*^ mice or BRG) mice [29, 30, 41].

NSG and BRG mice lack the γ_C_, whereas NOG mice have a truncated cytoplasmic domain of the gamma chain that binds to cytokines but lacks the signaling domain. These can be deployed for study using four general approaches to engraft a human immune system. The first model is the human peripheral blood leukocyte (PBL) severe immune deficiency (Hu-PBL-SCID) model which is generated by injection of human PBLs, where rapid engraftment of human CD3 + T cells occurs within one week. The model allows transient studies of human T cell function limited by the development of xenogeneic graft-versus-host disease (GVHD) [24]. The second model is the bone marrow/liver/thymus “BLT” model. This is generated by transplantation of human fetal liver and thymus under the kidney capsule and concurrent intravenous injection of autologous fetal liver hematopoietic stem cells (HSCs) [42, 43]. All lineages of human hematopoietic cells are developed, and the model supports a robust mucosal immune system. Human T-cells are educated in an autologous human thymus and are HLA-restricted. Despite these advantages, there are two major drawbacks including GVHD-like reactions [29, 30, 44] and limitations in obtaining fetal cells to generate the model. The third model is through the injection of human CD34 + HSCs derived from bone marrow (BM), umbilical cord blood, fetal liver, or granulocyte colony-stimulating factor (G-CSF)-mobilized peripheral blood. This model possesses BM-generated T cells, B cells, antigen-presenting cells (APCs), and myeloid cells, but are found at low levels. The human T cells are educated in mouse thymus and are H2 type, not HLA-restricted [45]. The fourth model is generated by intrahepatic injection of human CD34 + HSCs from human cord blood [26]. This model supports engraftment of a complete human immune system which lasts for more than one year with limited GVHD and is the most widely used due to reduced manipulation of the mice during their generation. The only disadvantage of this model is that the human T cells are educated in murine thymus and have functionally underdeveloped lymphatic tissues [46]. Despite these limitations humanized mice are commonly utilized as translational models in regenerative medicine, transplantation immunity, infectious disease research and for cancer biology and therapeutics.

## HIV-1 infection, pathogenesis, prevention, and antiretroviral testing

Species specificity of HIV initially precluded the use of mouse models for HIV infection; however, mice transplanted with functional human immune system (HIS) became a highly versatile and cost-effective model to study HIV-1 disease. Employment of humanized mice for HIV infection started when SCID mice were discovered [47]. Improvements in SCID mice strains have been made by refining the compatibility of mouse innate immune environment to allow human grafts. This has made it possible to have long-term reconstitution of the human immune system that supports chronic HIV infection. Humanized mice can induce adaptive immune responses and have been used, in measure, for vaccine testing [47, 48]. However, the human IgG responses are limited. This has been overcome by employing immunodeficient mice of different backgrounds with HSCs with thymus/liver tissue implants to generate BLT mice [49, 50]. Different human immune cell subset distribution in blood and lymphoid tissues allows BLT mice to be susceptible to HIV-1 infection. These mice can be infected through natural vaginal, rectal, or intravenous routes and used to study HIV-1 biology (viral entry, replication and spread), virus-induced immunopathology (CD4^+^ T-cell depletion and immune activation), mucosal inflammation, and cellular viral tropism [51–54]. HIV-1 reservoirs can also be established in infected humanized mice after treatment with combinations of antiretroviral drugs (ARVs), thereby providing a model to test new therapies for viral treatment and prevention. These mice can also be used to test how best to interrupt viral integration, activation, and replication [55, 56]. Recently our group employed humanized mice to examine tissue viral reservoirs and to recapitulate latent HIV-1 in vivo [57, 58]. These works demonstrated that mature macrophages are a cell reservoir in antiretroviral therapy (ART)-suppressed HIV-infected humanized mice [59]. Mice infected with HIV and treated with combination ART achieved complete viral suppression in the peripheral blood, and immune cells were sorted into T lymphocyte subsets and macrophages to quantify HIV RNA and DNA. While CD4 + memory cells were the principal T cell reservoir, integrated HIV-1 DNA was detected in the bone marrow and spleen macrophages. These findings were affirmed in humanized myeloid only mice (MoM) [60].

Existing antiviral medicines are designed to block essential steps of the virus life cycle. To gain access into the host cell, virus particles adsorb and bind to the CD4 and CCR5 or CXCR4 receptor and co-receptor proteins present on the host cell surface. Agents that block these interactions have been developed into effective drugs against HIV-1 [61]. Other antiviral drug targets include ion channel blockers or inhibitors of structural and non-structural viral proteins, reverse transcriptase enzyme, integrase, protease, and neuraminidase enzymes that catalyze polyprotein cleavage and release of mature virions. However, notable limitations include the narrow spectrum nature of the compounds, suboptimal adherence to daily regimens, poor bioavailability, drug resistance, and associated toxicities. The available antiviral agents are also virus-specific with only a few exhibiting broad-spectrum antiviral activities [62]. While development of broad-spectrum antivirals may seem to offer attractive alternatives to conventional target-specific antiviral drugs, their development into drug candidates has been hampered by either poor efficacy or toxicity concerns [63]. Limitations in delivery and failure to maintain therapeutic drug concentrations at sites of viral replication have also negatively impacted therapeutic outcomes. The absence of vaccines for chronic viral infections such as HIV and HCV has led to growing interest in long-acting (LA) formulations and devices aimed at improving patient adherence to therapy to minimize emergence of drug resistance [64, 65].

Humanized mice have been used to test newly developed and LA ART, neutralizing antibody, immunotherapeutic, latency re-activating agents, and viral gene editing strategies [17, 66, 67]. An example is the drug 4′-ethynyl-2-fluoro-2′-deoxyadenosine (EFdA), a nucleoside reverse transcriptase inhibitor (NRTI) that was developed in BLT mice. Works demonstrated that EFdA monotherapy was able to suppress viral replication [68]. Pre-exposure prophylaxis (PrEP) studies with EFdA prevented HIV-1 vaginal and oral transmission in BLT mice. Other LA ART studies were developed of medicines administered once a month. LA nanoformulated integrase inhibitor raltegravir protected humanized mice from repeated high-dose vaginal HIV challenges in a PrEP study [69]. Our own laboratories created LA nanoformulated protease inhibitors then tested them in humanized mice [70]. Nanoformulated atazanavir and ritonavir (nanoATV/r) combination suppressed plasma viral load below the detection level after six weekly doses, and ART cessation resulted in immediate viral rebound [71]. We also decorated nanoATV/r with folic acid for cell-specific targeting and uptake, and three doses given once every other week significantly improved viral suppression in chronically infected humanized mice compared to untargeted nanoATV/r [72, 73]. Next, we developed state of the art LA slow effective release (LASER) ART using prodrug technology with the ability to prevent or suppress HIV infection for a prolonged period after a single dose administration. New generation LASER ART formulations of cabotegravir and dolutegravir (CAB and DTG) could prevent and suppress HIV infection. Nanoformulated myristoylated CAB (NMCAB) after a single 45 mg/kg intramuscular injection, had pharmacokinetic (PK) profiles that were 4 times greater than that recorded for parenteral CAB. In mice, NMCAB showed significantly higher drug concentration up to one year after one IM injection as compared to current parenteral CAB formulations [74]. A hydrophobic and lipophilic modified DTG prodrug encapsulated into poloxamer nanoformulations protected humanized mice from the parenteral challenge of HIV-1 for two weeks [75]. Newer formulations of CAB prodrug nanoformulations have increased the apparent half-life of the drug to one year [76]. Humanized mouse models also demonstrate the antiviral effectiveness of broadly neutralizing HIV-1 antibodies [46]. It has been shown that a combination of antibodies can suppress viremia below the limit of detection and target the HIV-1 reservoir. Moreover, passively administered antibodies and vector-mediated expression of broadly neutralizing antibodies protect humanized mice from HIV-1 infection [77]. The mouse models also provide a potential bridge to predict immunotherapeutic-related cytokine release syndrome and development of HIV-1 cure strategies. PBLs from patients can be engrafted in adult immunosuppressed mice to study the response to immunotherapies, like anti-CD3, anti-CD28, Keytruda, anti-thymocyte globulin, and a TGN1412 analog.

## NeuroHIV and humanized mice

Soon after the discovery of HIV, it was found that mononuclear phagocytes (MP; monocytes, macrophages, microglia, and dendritic cells) are the principal viral targets within the central nervous system (CNS) [78]. HIV enters the brain during early stages of HIV infection, and the infected monocyte-macrophage act as Trojan horses in viral spread within the CNS [79]. For HIV-1 disease in the brain, MPs serve as viral reservoirs and inducers of end-organ disease and are the drivers of HIV-1 associated neurocognitive disorders (HAND), a clinical disease complex prevalent in up to 50% of infected people [80]. Introduction of ART has been effective in suppressing viral replication and reducing the severity of cognitive, motor, and behavioral impairments [81]. The virus persists in a latent form, and neither ART nor the host antiviral cellular and humoral immunity could eliminate infection leading to milder forms of memory impairments [82, 83]. Virus-induced MP functions lead to the production of cell and viral toxins that reflect an aberrant secretory immune response and persistent low-level infection [84]. Neuroinflammation through persistent glial infection and activation has emerged as a signature phenotype of HAND. Understanding the underlying molecular and cellular mechanisms in HAND pathology and viral persistence is essential to develop therapeutic strategies for HAND and for HIV elimination from CNS. While studies of the simian Immunodeficiency virus (SIV) have contributed to the current knowledge of HAND, the need for more scalable and affordable models lead to the initial development of a mouse model of neuroHIV in the 1990s [85]. Since HIV-infected MPs are the major drivers of HAND-associated pathology, human virus-infected monocyte-derived macrophages were injected intracerebrally into the caudate-putamen of immunodeficient mice [52, 86, 87]. Several important aspects of HIV-1 encephalitis (HIVE, the pathological equivalent of advanced virus-associated cognitive dysfunction) such as multinucleated giant cell pathology, activated microglia and astrocytes, myelin pallor, and dendritic loss were observed. Moreover, behavioral, and cognitive abnormalities in the HIVE mice were associated with neuronal dysfunction and decreased synaptic density. The HIVE mouse model has been used to test anti-inflammatory, antiretroviral, or neuroprotective therapeutic approaches [86]. Initial studies in this model provided a direction in understanding efficient ART regimens to suppress viral load in the HIV infected brain.

Further improvements to include the adaptive immune component of HIV infection in neuroHIV was achieved by the reconstitution of immunodeficient animals with syngeneic human peripheral blood lymphocytes followed by intracranial injection of HIV-1-infected macrophages resulted in cytotoxic antivirus T lymphocyte (CTL) response [88]. CD8-positive T cells migrated to the sites of human macrophages leading to the cell-mediated destruction of HIV-1 infected cells. Development of HIVE mice reconstituted with a human immune system enabled testing of immunomodulators that included indoleamine 2,3-dioxygenase (IDO) inhibitors, peroxisome proliferator-activated receptor (PPAR) gamma, and cannabinoid 2 (CB2) receptor agonists [88]. The major limitations of the HIVE mouse models are associated with traumatic injury caused by the cell injections into the brain, focal neuropathology around the injected area and an imperfect relationship between the brain neuropathology and progressive systemic infection. Moreover, the HIVE and AIDS dementia complex was prevalent during pre-ART era and modeling milder forms of HAND requires mice that can be chronically infected with HIV and with suppressive ART.

Mice reconstituted with human immune system allowed to study chronic HIV infection, however, a limitation in humanized mouse models involves the distribution of human cells in the brain. Human cells are located mainly in the meninges; with very few in perivascular areas and brain parenchyma. Moreover, human microglial-like cells are rarely found in the mouse brain. HIV-infected human macrophages and lymphocytes are mainly found in meninges and perivascular areas [87, 89, 90]. Longitudinal non-invasive imaging studies using diffusion tensor imaging (DTI) and magnetic resonance spectroscopy (MRS) revealed progressive loss of neuronal integrity, which correlated with gliosis and loss of neuronal dendritic and synaptic proteins and myelin [91]. Behavioral abnormalities such as memory loss and anxiety were also observed in HIV-1 infected humanized mice [91]. HIV related behavioral deficits were mostly studied in non-humanized rodent models, including HIV transgenic rodents [92–97] and EcoHIV infected mice [98–100].

Humanized MoM reconstituted with human myeloid and B cells, but no T cells, showed productive infection of HIV-1 in MPs and led to the viral seeding in CNS by infected MPs [60]. Presence of both classical and intermediate macrophages were observed in the brains of MoM, but the lack of human microglia limited productive brain infection. In a humanized T cell only mouse, T cells could also establish and maintain HIV infection in the CNS [101]. Nonetheless, in all the humanized mouse models, HIV brain infection is minimal due to the limited number of human cells [102].

**Fig. 1:**
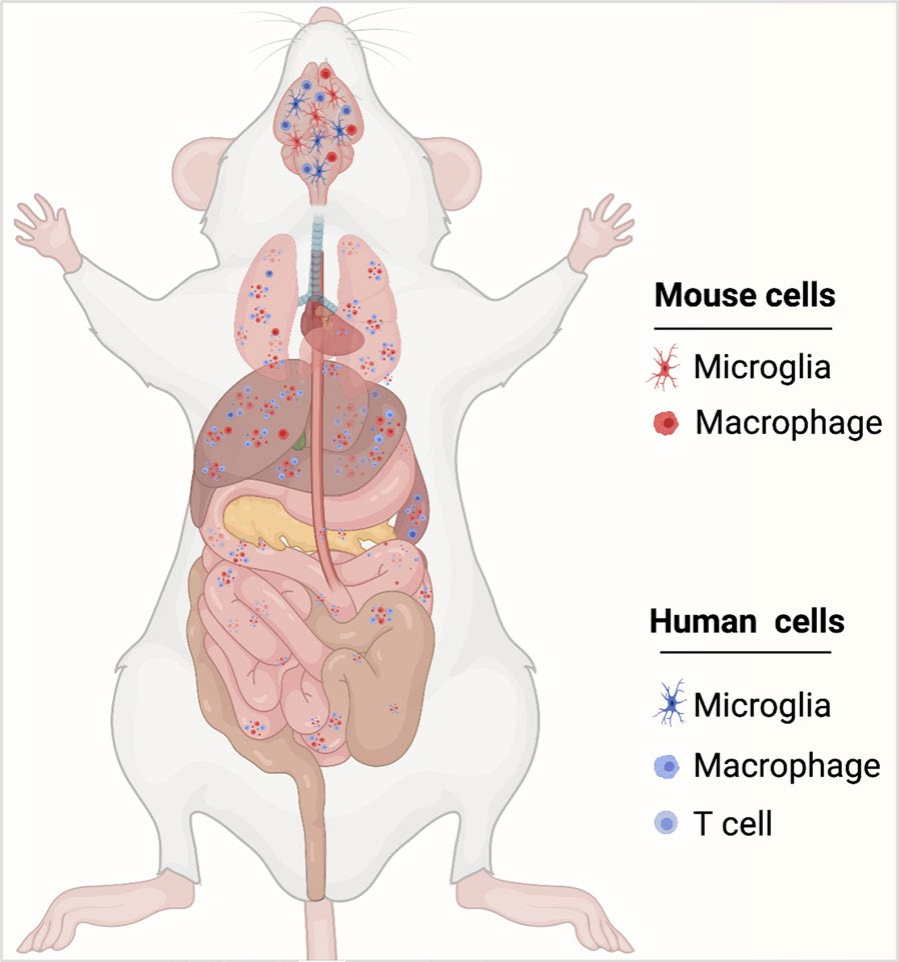
Development and characterization of humanized microglial mice. Humanized microglia mice serve as tool to elucidate neuroHIV pathobiology and to develop therapeutic and elimination antiretroviral strategies for HIV infections. IL-34 was transgenically introduced and at the same time CD34 + HSCs were injected into the liver of immunodeficient newborn mice. These mice developed a functional human lymphoid system comprised of B cells, T cells and macrophages and brain enriched with human microglial cells. Blue and red colored cells are human and mouse cells, respectively. In regards to the timing, human cells are introduced after birth to reconstitute lymphoid and solid organ tissues devoid of endogenous murine immunocytes. The human immunocytes show limited number reductions with time after injection as new cells are produced from progenitors. These exist, over time, less frequently in brain and periphery compared to mouse cells

As noted, MPs are the major cellular targets for HIV-1 in brain, human astrocytes can be infected but at very low levels [103, 104]. Both microglia and astrocytes contribute to the CNS viral reservoir and neuroinflammation. To utilize the humanized mice for neuroHIV studies, the presence of human glia in the mouse brain along with the human immune system are necessary. The ability to reconstitute the murine brain with functional human glial cells would provide an opportunity to study HIV induced inflammation, neuronal dysfunction, and viral reservoirs in one system. Our laboratories generated a humanized mouse model dually reconstituted with human astrocytes and human leukocytes [105]. By transplanting human neuroprogenitor cells in the brain and HSC in the liver simultaneously in a new-born mouse, led to the development of human astrocytes and leukocytes. In these mice human glial-specific anti-viral response was observed following systemic HIV infection, and the neuropathogenesis was observed as downregulation of mouse genes crucial for oligodendrocyte differentiation and myelination, suggesting alterations in structure and function of white matter. HIV brain infection was minimal in this model, again restricted to macrophages and lymphocytes in meninges, and very few perivascular and parenchymal human leuokocytes, due to the lack of human microglial reconstitution. To facilitate human microglial differentiation in HSC-transplanted humanized mice, IL-34, a tissue specific ligand for colony stimulating factor-1 receptor (CSF-1R), was transgenically introduced into immunodeficient mouse strain (Fig. 1). IL-34 is important for human microglial and tissue macrophage development. Human HSC reconstitution in human IL-34 transgenic immune deficient mice lead to the engraftment of a mouse brain with human microglia that expressed canonical markers such as CD14, CD68, CD163, CD11b, ITGB2, CX3CR1, CSFR1, TREM2, and P2RY12 [106]. Peripheral HIV infection led to productive infection of human microglia with a significant number of HIV-1 antigen positive cells distributed in all mouse brain regions. Human-specific molecular signatures representative of antiviral and neuroinflammatory responses were detected. Transcripts for all viral proteins were readily identified with the highest expression of HIV env, pol and nef. Further, neuropathological assessments during HIV infection are under investigation. Our recent studies using human microglia mouse model demonstrated significant levels of HIV-1 DNA in the brain and other lymphoid tissues even under combination ART controlled viral infection supporting the establishment of CNS viral reservoirs in mice. These improved humanized glial mouse models permit investigations of neuroHIV in presence of suppressive ART. Further studies of HIV induced neuropathology and behavioral deficits in HIV infected and ARV treated humanized microglial mice will provide a better understanding of the human disease and the underlying molecular mechanisms for successful therapeutic development. This new model to study HIV brain infection also aid in the development and testing of new generation ART delivery with improved CNS bioavailability and will be useful for future viral eradication studies. Further, the model now allows studies of newly emerging ART-induced neurotoxicity such as reported for efavirenz [107, 108]. However, many laboratory and animal studies have shown a number of direct effects on neuronal and glial function along with pathological outcomes that are linked to amyloid deposition, small vessel damage and aberrations in chemical neurotransmission [109–114]. Studies of macrophage function as part of depot for sustained release agents are now possible with these newer humanized mouse models [59, 105, 115–118]. Perhaps ever more important rests in the need of vigorous behavioral testing which have been initiated in the earlier models but remain underdeveloped in these humanized microglial brain test systems [106, 119].

## Hepatitis B

It is estimated that approximately two billion people worldwide have evidence of past or present infection with hepatitis B virus (HBV), and 257 million individuals are chronic carriers (i.e., positive for hepatitis B surface antigen [HBsAg]). The rate of progression from acute to chronic HBV infection (CHB) is approximately 90% for perinatally-acquired infection, however vaccination has reduced the progression by 90% [120]. A significant proportion of people living with HIV-1 are also infected with HBV [121, 122]. However, the number of existing CHB patients exceed number of people living with HIV-1 [64]. The progression of CHB leads to the development of cirrhosis and hepatocellular cancer [123]. CHB remains a significant burden on health care system around the world and requires effective treatment to prevent progression [21]. The goal for CHB patients is to achieve a cure, however the complexity of the viral life cycle and multiple mechanisms of avoidance of immune responses cause complications. The formation of stable covalently closed circular DNA (cccDNA) as a replication template of HBV also represents a significant challenge for elimination. The elimination of hepatocytes with integrated HBV genome is immune mediated and required for clearance of HBsAg. All steps in HBV lifecycle are present in human hepatocytes, and humanized mice are an instrumental tool to evaluate the efficacy and safety of available therapeutics. Several models are reported to humanize the mouse liver and establish HBV infection [124]. Human liver chimeric mice are often generated using the urokinase-type plasminogen activator transgene (uPA) and RAG-2 gene knockout (uPA/RAG2^−/−^) mice[125]; uPA/SCID [126], mice deficient in the tyrosine catabolic enzyme fumarylacetoacetate hydrolase (Fah^−/−^) on Rag2^−/−^ interleukin 2 receptor gamma chain knockout (Il2rg^−/−^) mice (FRG) [127], and herpes simplex virus type-1 thymidine kinase- NOD/Shi-scid IL2r-gamma(null) NOG (TK-NOG) mice [128]. Different levels of liver humanization can be achieved in these models and different strains of HBV (and hepatitis delta virus) that can naturally infect human hepatocytes. The established chronic HBV viremia (10^5^–10^10^ IU/mL HBV DNA) and HBsAg stable expression presence in circulation, are used to monitor treatment efficacy and liver tissues for evaluation of cccDNA copies. The use of chimeric mice for anti-HBV therapeutics are described in detail [129].

Existing treatment of CHB is based on inhibition of viral RNA reverse transcription to prevent replenishment of cccDNA by nucleot(s)ides (NAs), which requires a life-long administration of oral drugs with strong adherence. Entecavir remains the most used oral therapeutic in humanized mice experimental combinatorial treatments [130]. LA lamivudine nanoformulation was developed by our laboratories and tested on humanized mice. A single intramuscular injection of 75 mg/kg reduced HBV DNA in peripheral blood for up to 2.5 log for 4 weeks [115]. The differences of HBV genotypes and drug-resistant mutants (to entecavir and lamivudine) susceptibility to 90 mg/kg body weight/day of TDF for 3 weeks were tested on uPA/scid mice [131]. The effects of NAs to inhibit reverse transcription and HBV DNA synthesis and antiviral properties of IFN-α showing enhanced cccDNA degradation were fully reproduced in humanized liver mice [132]. IFN-α-mediated suppression of HBsAg concentration and silencing of cccDNA was extensively studied on uPA/scid and uPA/scid/IL2Rgc-/- (USG) liver humanized mice [133, 134]. The effective new approaches targeting HBsAg that tolerates the immune system and support viral persistence were assessed in humanized liver mice. ARB-1740 is a clinical stage RNA interference agent composed of three siRNAs delivered using lipid nanoparticle technology (LNP). A combination of ARB-1740 with a capsid inhibitor and pegylated interferon-alpha led to greater liver HBsAg reduction which correlated with more robust induction of innate immune responses in cDNA-uPA/scid human chimeric mouse model of HBV [135, 136].

The lipid nanoparticles (LNPs) containing HBsAg silencing RNA were modified with a hepatocyte-specific ligand, N-acetyl-d-galactosamine (GalNAc) and tested on chimeric uPA/scid mice [137]. Modification of the GalNAc-LNPs with polyethyleneglycol negated the LNP-associated toxicity without any detectable loss of gene silencing activity in hepatocytes. A single injection of the modified LNPs resulted in a significant reduction of HBV genomic DNA and their antigens [137]. Multiple approaches targeting capsid proteins were tested on chimeric humanized mice. For example, ciclopirox, a synthetic antifungal agent, inhibits HBV capsid assembly and secretion of HBV DNA in infected liver chimeric uPA/scid mice alone or synergized by Tenofovir disoproxil fumarate (TDF) (six weeks orally) [138]. GLP-26, a novel glyoxamide derivative that alters HBV nucleocapsid assembly and prevents viral DNA replication, in combinatorial treatment with entecavir in a humanized mouse model showed reduction in viral load and viral antigens, which was sustained for up to 12 weeks after treatment cessation [139]. Humanized uPA/scid mice were also used to evaluate NVR3-778, a capsid assembly modulator, in combination with PEG-IFN, and showed positive effect as compared with entecavir [140]. The adeno-associated virus (AAV) vectors and CRISPR-*Staphylococcus aureus* (Sa)Cas9 were used to edit the HBV genome in liver-humanized FRG mice chronically infected with HBV and treated with entecavir, which showed reduction in total liver HBV DNA and cccDNA [141]. Similar studies of anti-HBV effects of the AAV2-/WJ11-Cas9 system in a uPA/scid humanized chimeric mouse model also showed reduced HBV infection [142].

Humanized liver mice were also used to assess the efficacy of cellular immune-mediated elimination of HBV infected human hepatocytes. For example, transplantation of USG mice with human HLA-A2-positive hepatocytes enables testing of cytotoxic T lymphocyte-mediated activity. The engineered T cell receptors recognize HBV core and HBsAg-derived peptides and then eliminate HBV infected cells reducing viremia [143]. This approach was tested in combination with Myrcludex B, which prevents HBV entry. The adoptive transfer of PreS1 antibodies prevented, or modulated, HBV infection after a subsequent challenge of the virus in humanized uPA/scid mice for 3 to 8 weeks [144]. In addition to listed complex approaches, long-acting formulations of existing NAs with activities against HBV have great potential to end the HBV epidemic, and humanized mouse models are better suited for the advancement in studying such formulations.

## Viral cure strategies and humanized mice

Viral infections constitute a major public health threat that underscore the need for innovative approaches and preparedness to combat pandemics. Treatments with antiviral drugs are used to limit the severity of illness without eliminating the virus from the host cells. While vaccines would be ideal in combating infections, rapid viral mutations and heterogeneity have posed significant challenges with only a few effective vaccines available for a limited number of viruses [61]. For instance, the high genetic variability and immune escape exhibited by HIV and other RNA viruses such as HCV have impeded the development of safe and effective vaccines against all types and subtypes of the pathogen. These limitations highlight the need for development of effective interventions that target multiple replication pathways to be tested in appropriate animal model systems.

**Fig. 2:**
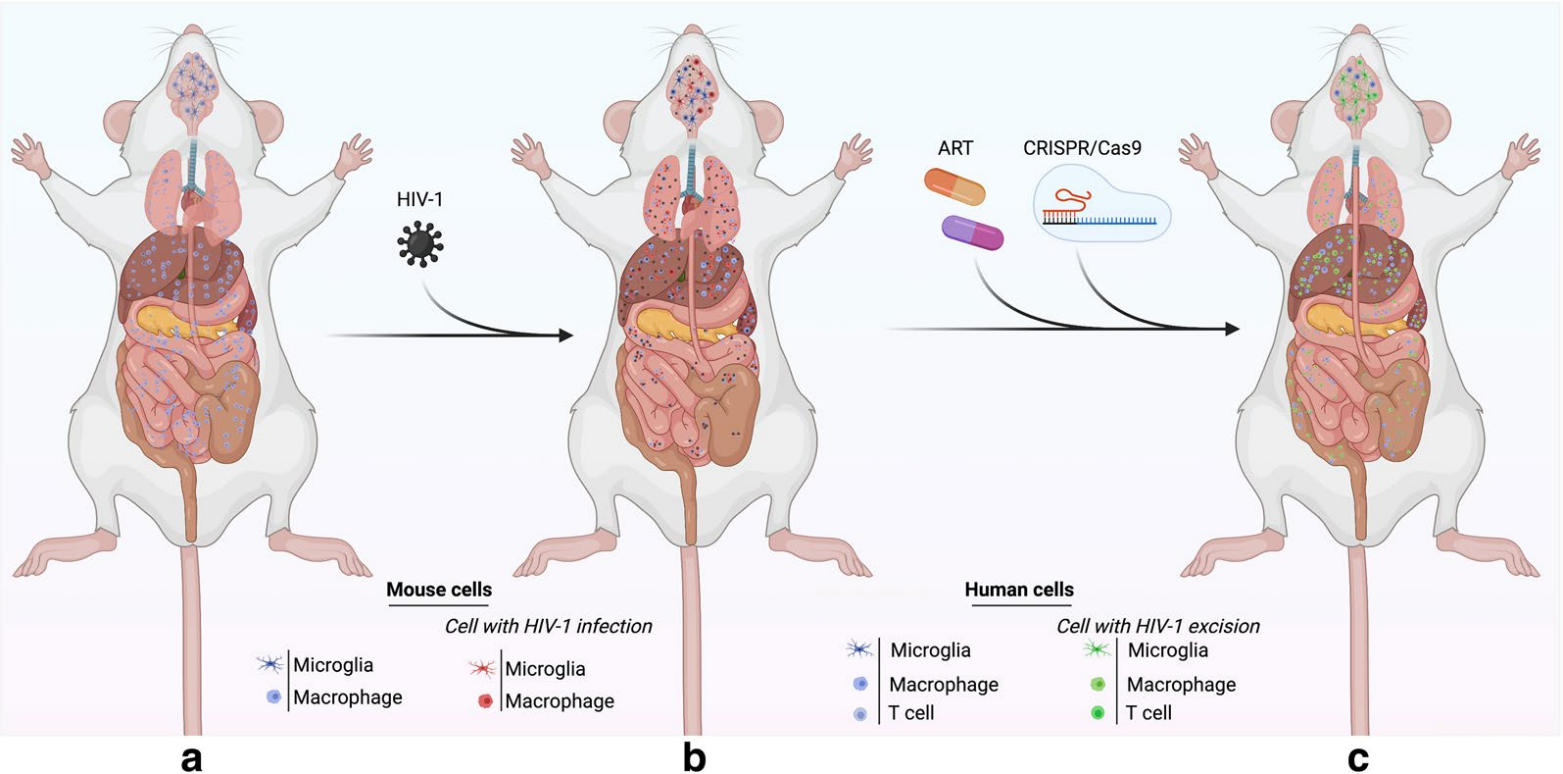
LASER ART and CRISPR-Cas9 therapies for HIV-1 elimination. Humanized mice developed (**a**) and HIV-1 infected humanized (**b**) mice were administered with sequential treatment of a combination of long-acting ART followed by CRISPR-Cas9 targeting specific sequences of the HIV-1 genome (**c**). Using sequential treatments, complete HIV-1 elimination from a subset of animals. This combinatorial approach is being developed for improved delivery of CRISPR-Cas9 to target the latent reservoirs in humanized mouse models (left panel), to improve the rates of viral elimination (right panel) (Color explanation: blue color cells are represented as human cells, red color cells are represented HIV-1 infected cells and green color represents the cells with complete elimitation of HIV-1)

CRISPR-Cas based genome editing represent a novel tool that has wide-ranging applications in the treatment of various infectious and neurodegenerative diseases [145, 146] and can be used to insert, delete or modify target genes with very high precision and accuracy [147, 148]. CRISPR Cas allows for precisely edited mouse models and opens doors of unlimited possibilities. CRISPR-Cas can be used in humanized mouse models to advance the treatment of diseases like cancer, diabetes, viral and nonviral infectious diseases. Hemophilia A patient-derived pluripotent stem cells were edited ex-vivo using CRISPR and then transplanted into the hind limb of hemophilia mice, increased survival of the mice was observed [149]. Similar approaches have been employed for other hematological abnormalities. The ability of CRISPR to make edits ranging from a single base to the insertion of long sections of DNA opens the door for humanized mouse models where mouse genes are replaced with human genes at multiple loci [150] and will improve humanized mouse models for neurodegenerative diseases [151]. Using a combination of LASER ART and CRISPR-Cas9 HIV excision strategies, our group recently achieved HIV eradication in a subset of HIV infected humanized mice [152]. This is the first study of its kind demonstrating HIV elimination from infected animals (Fig. 2). CRISPR-Cas9 has been proposed as a means of mimicking the CCR5 delta 32 mutations that provides a small percentage of the human population resistance against HIV infection. Human primary CD4^+^ T cells were expanded then transduced with lentivirus delivering CRISPR-Cas9 against CCR5. After the CCR5 modification was confirmed, these cells were transplanted in a NOD-Prkdc^em26Cd52^Il2rg^em26Cd22^/Nju mice and reconstituted mice challenged with HIV-1. These animals displayed some degree of resistance but failed to provide complete protection against HIV [153]. Use of preclinical mouse models and proper screening can provide a better solution to study infectious diseases and to find cure.

## Neurodegenerative diseases and future employments of humanized mice

### Alzheimer’s disease

Alzheimer’s disease (AD) is the most common neurodegenerative disease affecting the elderly population and is the sixth leading cause of death in the United States [154, 155]. Promising outcomes in preclinical studies have not always yielded positive clinical outcomes [156]. Recent advancements have revealed that current animal models lack important biological features and therefore are unable to mimic human disease pathology precisely. AD researchers have commonly used first-generation transgenic mouse models that overexpress proteins linked to familial AD, mutant amyloid precursor protein (APP), or APP and presenilin. While these mice can demonstrate AD pathology the animal models lack important biological features and therefore are unable to mimic human disease pathology precisely. This has given rise to second-generation mouse models which contain humanized sequences and clinical mutations in the endogenous mouse *App* gene. Thus, limitations of first-generation animal models are now successfully overcome by the development of humanized knock-in mice as second-generation models [157]. Human and mouse immune and neuronal cells are different at the transcriptional levels, therefore, offer differential responses against AD pathological proteins, which can affect the efficacy of therapeutic candidates in clinical testing [158, 159]. With field advancement, human-induced pluripotent stem cells have been transplanted into the mouse brain, allowing for study on how amyloid pathology affects human neurons in the context of a multicellular brain environment [160]. Additionally, transplantation of human-induced pluripotent stem cells into immunodeficient mice allowed development of human microglia cells, which interact with Aβ differently compared to the other humanized mouse counterpart [161]. Our laboratory recently developed human IL-34 transgenic mice under immunodeficient genetic background, where upon transplantation of human hematopoietic stem cells resulted in human-like microglia cells development in the brain [106]. The human adaptive immune response is different from those mice [162, 163], which further affects APP expression and Aβ accumulation. Therefore, with the emerging role of the innate and adaptive immune arm in AD pathogenesis and their differential regulation in two different species, the urgent development is needed for better small animal models with the complete human immune system.

### Parkinson’s disease 

The defining characteristic of Parkinson’s disease (PD) is the progressive loss of dopaminergic neurons originating in the substantia nigra (SNpc) and innervating to the striatum resulting in the concomitant loss of dopamine, the principal movement-controlling neurotransmitter [164, 165]. This loss leads to the progressive development of primary motor dysfunction and deficits, including resting tremor, bradykinesia, muscle rigidity, and postural instability. PD hallmarks include neuronal Lewy body inclusions that are comprised primarily of misfolded, oligomerized α-synuclein (α-syn), and histological evidence of neuroinflammation as indicated by reactive microglia encompassing regions of α-syn aggregation and neurodegeneration [166–169].

**Fig. 3:**
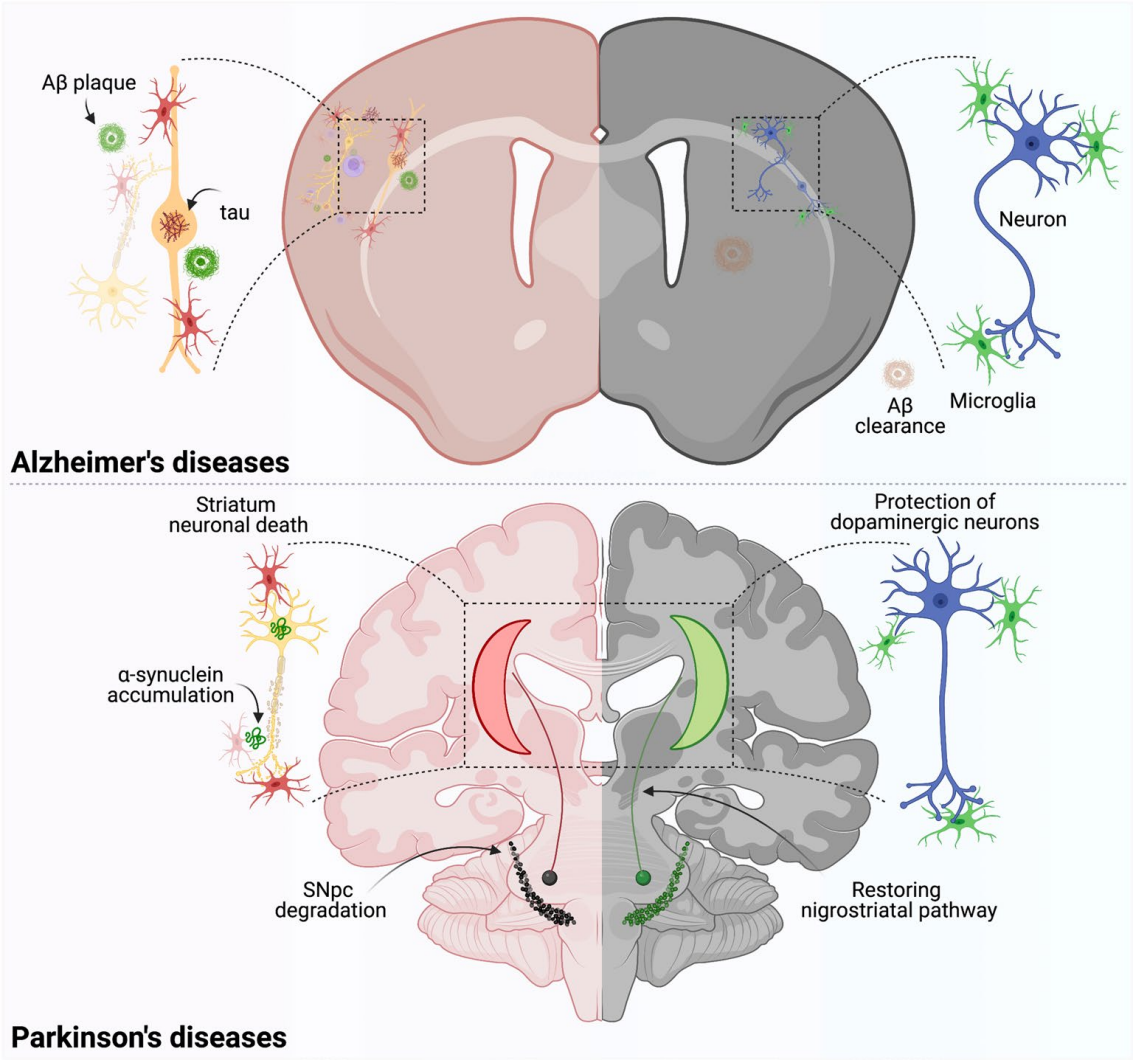
Immune transformation in neurodegenerative disorders. Pathogenic changes seen in the brains of AD include accumulation of intraneuronal neurofibrillary tangles of Tau and extracellular Aβ plaques. These induce activation of CNS resident microglia and astrocytes leading to neuroinflammation (top left). In contrast, transformation of an inflammatory microglia by medicines or immune modulation leads to neuronal protection and maintenance of CNS homeostasis (top right). Similarly in PD, aggregated self-protein α-synuclein activates microglia leading to neuronal damage within the substantia nigra pars compacta along with their connections into the striatum; brain subregions responsible for coordinate movement (bottom left). However, brain homeostasis achieved through neuroprotection (bottom right) can affect clinical improvements

Rodent models of PD have been utilized to evaluate immunomodulatory agents that target various inflection points along the neuroinflammatory pathway. However, whether pro-inflammatory models that do not include human components as targets will provide sufficient robustness to bring translational therapeutics to completion has been contentious. To determine the acuity of the human immune system in a PD model, NSG mice were reconstituted with human CD34 + HSCs, and were considered engrafted with at least 25% HuCD45 + peripheral mononuclear cells (PMNCs) by 12 weeks post-reconstitution [170]. Engrafted mice and age-matched wild type mice were treated with three doses of 1-methy-4-phenyl-1,2,3,6-tetrahydropyridine (MPTP), a neurotoxin known to cause PD like symptoms, at 18 mg/kg/dose every two hours. One MPTP-treated group from each strain was treated with tacrolimus (FK506), a calcineurin/NFAT inhibitor that suppresses T-lymphocyte signal transduction pathways and IL2 transcription and is indicated for organ transplantation and ulcerative colitis [171, 172]. Previous studies demonstrated that FK506 reduced α-syn aggregation and microglial activation with subsequent neuroprotection in animal models of PD, including MPTP- and α-syn overexpression-induced dopaminergic neurodegeneration [173–175]. Utilizing MPTP, this study provided the first demonstration of induced PD-like lesions and motor deficits in humanized CD34 + mice [170]. Of note, treatment of humanized mice with MPTP/FK506 resulted in enhanced survival of dopaminergic neurons in the SN and efferent striatal termini, whereas less survival was demonstrated in MPTP/FK506-treated wild type mice. As expected, MPTP increased levels of human cytokines, and FK506 treatment diminished levels of most human cytokines in plasma from MPTP-treated humanized mice, whereas only levels of human IL-4, IL-6, IL-8, and IL-12 were diminished from striatal tissues. Interestingly, FK506 treatment did not significantly affect levels of mouse cytokines from plasma or striatum compared to MPTP-treated wild type animals. This suggests that the humanized mouse platform may provide a more robust model for evaluation of translatable therapeutics in rodent models of PD. However, it should be noted that few CNS-infiltrating macrophages/microglia were of human origin as evidenced by low levels of expression of HuCD45, HLA-DR, and human CD68 in the CNS compared to the peripheral tissues. Additionally, the strains of the mice were of disparate backgrounds with humanized mice derived from the NOD/ShiLtJ compared to C57Bl/6 mice as wild types. Moreover, this study utilized female mice, whereas most MPTP studies are performed in male mice to reduce known variability observed with females. With the advent of ART for HIV-1 treatment, individuals living with HIV have longer lives with fewer co-morbidities, yet still can exhibit motor deficits like PD [176–180]. Thus, a major, yet recurrent question is whether HIV infection affects the development of PD-linked neurodegeneration. To address that question, we investigated the effect of HIV infection on nigrostriatal dopaminergic neurodegeneration [181]. We initially used NSG mice reconstituted with CD34 + human HSCs that could engraft for 18 weeks to attain humanized mice with high levels of CD45 + cells. Humanized mice were infected for three weeks with HIV-1 prior to intoxication with four doses of MPTP at 14 mg/kg/dose every two hours. This study showed that in the MPTP model, acute HIV infection afforded no discernable susceptibility to dopaminergic neurodegeneration as demonstrated by insignificant differences of tyrosine hydroxylase (TH) + neuronal loss in the SN that ranged from 13 to 27% and losses of striatal termini of 46% to 53%. Moreover, levels of microglia from HIV/MPTP were not significantly elevated regardless of HIV infection duration. Thus, these findings indicated either the lack of a synergistic effect, lack of interaction between the reconstituted human lymphocytes and murine microglia within the humanized system, or the inability of HIV to sufficiently infect mouse microglial cells. The limited loss of dopaminergic neurons is most likely due to the initial neurotoxicity associated with MPTP, rather than the ensuing inflammatory cascade linked to immune activation. Therefore, lack of a neurodegenerative phenotype associated with MPTP use in humanized, male mice may indicate the need for experimentation in a different rodent model of PD, such as α-syn overexpression or the requirement of better CNS reconstitution of human microglial cells. As novel therapeutic strategies are developed, humanized animal models of neurodegenerative diseases (Fig. 3) are strongly needed to accelerate translation from preclinical to clinical setting [106, 182, 183]. There are obvious strengths, restrictions and opportunities of modeling functional and behavioral deficits associated with neurodegenerative disorders using humanized mice models*. *First, when fully developed such models would allow investigations of functional neuronal defiicts that link to behavioral outcomes in the context of a functional human immune system. Second, neurological disorders may be modeled more exactly as the role of both innate and adaptive immunity comes more significant in disease pathobiology. Third, a clear understanding of the role human immunity plays at the neurobiochemical levels can be uncovered and especially those that predict behavioral insufficiencies and vice versa. Especially in the case of PD where gait and locomotor abnormalities have been well-characterized in prior rodent models these can now be fully explored in the context of human T cell functions and immune tolerance [170, 184–187]. While motor deficits in the humanized CD34 + mice have been described behavioral comparisons between established rodent models and humanized models await future studies in these exciting models reflective a broad range of human infectious and degenerative diseases [186].

## Conclusions

**Fig. 4:**
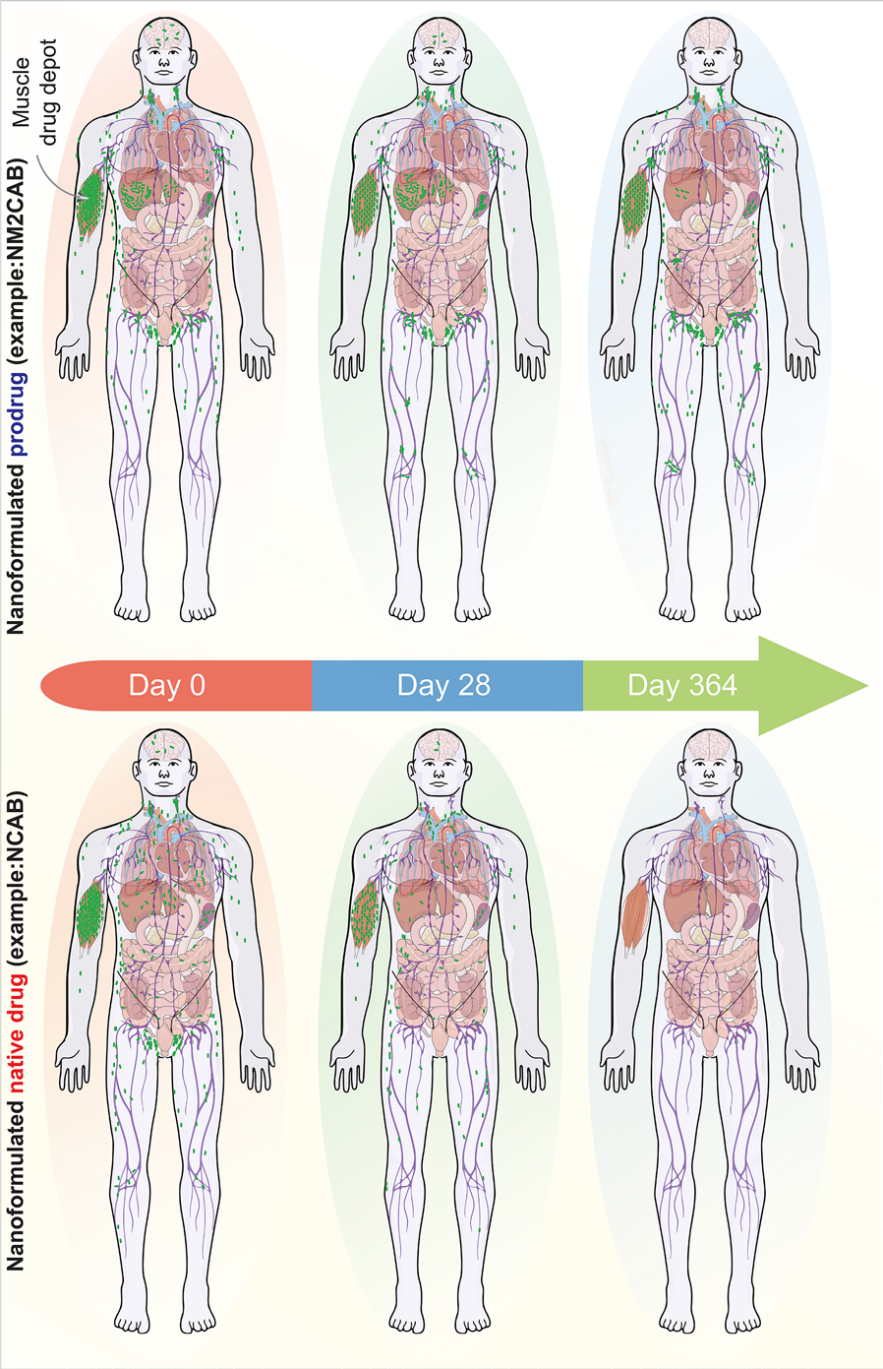
Potential of LASER ART for HIV-1 treatment and prevention. Based on extensive animal modeling, a single intramuscular injection of a nanoformulated stearoylated CAB ester prodrug (NM2CAB) can lead to sustained drug levels at the site of injection and within the reticuloendothelial system for up to one year (top panel). The formed CAB nanocrystals are absorbed from the injection site and undergo dissolution for prodrug that is subsequently hydrolyzed into active CAB in blood and tissue. CAB prodrug was recorded in all the tissues during a year-long observation in rodents and rhesus macaques after a singleNM2CAB injection. Top panel reflects how NM2CAB can be distributed after an intramuscular injection. By contrast, the nanoformulated NCAB is rapidly cleared from the site of injection and tissues. For NCAB, therapeutic drug levels are present in lymphoid tissues for one month (bottom panel)

Humanized mice represent the mainstream of available small animal models used to reflect the pathobiology and developmental therapeutics for human infectious, GVHD, cancerous and degenerative diseases. CD34 mouse models are employed in a variety of platforms seeking drug safety and efficiency and especially those that can modulate the immune system. Altogether, exhaustive research performed from multiple laboratories continues to identify and develop novel disease-modifying treatment options for viral, non-viral and neurodegenerative disease. The pace of the therapeutic development strongly relies on the quality and optimization of preclinical models. Hence, such models can ensure improved translation of various hopeful preclinical results into interventions that will ultimately benefit patients. To this end, we are pleased to provide an example from our own laboratories in the field of LA ART. Herein humanized mice were used to test efficacy, safety, and pharmacokinetics that have sped the development of our year long NM2CAB nano formulation. From these early works in mice, we were able to decipher dose, biocompatibility, cell and tissue drug distribution, immune responses, dissolution parameters and antiretroviral effectiveness. For the NM2CAB we found the prodrug nano formulation to be sustained in a muscle depot then disseminate to the lymphoid system and solid organs with slow-release rates that lead to an extended half-life. Phase 1 clinical trials are now being planned because of these early mouse experiments that facilitated development and safety of these new formulations (Fig. 4). However, this is yet one single example of the promise of human translation by having the ability to mimic human disease processes in a small animal. To that the best is still yet to come.

## Data Availability

Not applicable.

## Abbreviations

RT: Reverse transcriptase
HTLV: Human T cell leukemia virus
ART: Antiretroviral therapy
FDA: Food and Drug Administration
HIV: Human Immunodeficiency virus
NNRTI: Non-nucleoside reverse transcriptase inhibitor
NRTI: Nucleoside reverse transcriptase inhibitors
PR: Protease
ISNI: Integrase Strand Transfer Inhibitor
CAB: Cabotegravir
DTG: Dolutegravir
